# Prediction of 28-day mortality in critically ill patients with COVID-19: Development and internal validation of a clinical prediction model

**DOI:** 10.1371/journal.pone.0254550

**Published:** 2021-07-13

**Authors:** Matteo Luigi Giuseppe Leoni, Luisa Lombardelli, Davide Colombi, Elena Giovanna Bignami, Benedetta Pergolotti, Francesca Repetti, Matteo Villani, Valentina Bellini, Tommaso Rossi, Geza Halasz, Serena Caprioli, Fabrizio Micheli, Massimo Nolli

**Affiliations:** 1 Department of Anesthesia and Intensive Care Unit, Guglielmo da Saliceto Hospital, Piacenza, Italy; 2 Unit of Interventional Pain Management, Guglielmo da Saliceto Hospital, Piacenza, Italy; 3 Unit of Operating Room and Waiting Lists Management, Guglielmo da Saliceto Hospital, Piacenza, Italy; 4 Department of Radiological Functions, Radiology Unit, Guglielmo da Saliceto Hospital, Piacenza, Italy; 5 Unit of Anesthesiology, Division of Critical Care and Pain Medicine, Department of Medicine and Surgery, University of Parma, Parma, Italy; 6 Department of Morphology, Surgery and Experimental Medicine, Section of Anesthesia and Intensive Care Unit, University of Ferrara, Ferrara, Italy; 7 Cardiology Department, Guglielmo da Saliceto Hospital, Piacenza, Italy; 8 Controller and data management of Administrative Department, Guglielmo da Saliceto Hospital, Piacenza, Italy; Kaohsuing Medical University Hospital, TAIWAN

## Abstract

**Background:**

COVID-19 pandemic has rapidly required a high demand of hospitalization and an increased number of intensive care units (ICUs) admission. Therefore, it became mandatory to develop prognostic models to evaluate critical COVID-19 patients.

**Materials and methods:**

We retrospectively evaluate a cohort of consecutive COVID-19 critically ill patients admitted to ICU with a confirmed diagnosis of SARS-CoV-2 pneumonia. A multivariable Cox regression model including demographic, clinical and laboratory findings was developed to assess the predictive value of these variables. Internal validation was performed using the bootstrap resampling technique. The model’s discriminatory ability was assessed with Harrell’s C-statistic and the goodness-of-fit was evaluated with calibration plot.

**Results:**

242 patients were included [median age, 64 years (56–71 IQR), 196 (81%) males]. Hypertension was the most common comorbidity (46.7%), followed by diabetes (15.3%) and heart disease (14.5%). Eighty-five patients (35.1%) died within 28 days after ICU admission and the median time from ICU admission to death was 11 days (IQR 6–18). In multivariable model after internal validation, age, obesity, procaltitonin, SOFA score and PaO_2_/FiO_2_ resulted as independent predictors of 28-day mortality. The C-statistic of the model showed a very good discriminatory capacity (0.82).

**Conclusions:**

We present the results of a multivariable prediction model for mortality of critically ill COVID-19 patients admitted to ICU. After adjustment for other factors, age, obesity, procalcitonin, SOFA and PaO2/FiO2 were independently associated with 28-day mortality in critically ill COVID-19 patients. The calibration plot revealed good agreements between the observed and expected probability of death.

## Introduction

Coronavirus disease 2019 (COVID-19) is a primarily respiratory tract infection caused by a newly recognized betacoronavirus named SARS-CoV-2, firstly diagnosed in China (Wuhan), in December 2019 [1]. Since then, the outbreak of this infection has spread rapidly across the globe. As of March 15, 2021, 87.994 critically ill patients and 2.668.036 deaths had been reported [2]. The clinical spectrum of COVID-19 ranges from asymptomatic infection to severe respiratory failure [3].

Piacenza is a small city of Northern Italy very close to Codogno, the first city where a COVID-19 patient was identified in Italy. Consequently, the local hospital was quickly changed in a "COVID-19 hospital" [4] to manage a sudden increase in COVID-19 patients requiring hospital admission.

The knowledge of COVID-19 patient characteristics and risk factors associated with intensive care unit (ICU) admission and mortality is still limited. Older age, male sex, comorbidities, lower ratio of arterial partial pressure of oxygen/fraction of inspired oxygen (PaO_2_/FiO_2_) and higher SOFA score (Sequential Organ Failure Assessment) are independently associated with worse outcome in those admitted to the ICU [5–7].

However, only few studies analyzed the clinical characteristics and predictors of mortality in COVID-19 patients admitted to ICU in Italy [8, 9]. The definition of risk factors for mortality are mandatory to guide ICU capacity and resource allocation.

The aim of this study was to develop a clinical prediction model for 28-day mortality in critically ill COVID-19 patients admitted to ICU.

## Material and methods

### Population

This study was approved by the Local Ethics Committee and was conducted at Guglielmo da Saliceto Hospital of Piacenza. We retrospectively analysed a cohort of consecutive critically ill patients admitted to our ICU from Feb 22, 2020 to Apr 3, 2020 diagnosed with SARS-CoV-2 pneumonia, according to WHO interim guidance [10]. All the data were fully anonymised before the access and a random alphanumeric code was used to identify each patient in the database.

COVID-19 infection was diagnosed by a positive result of real-time reverse transcriptase–polymerase chain reaction (RT-PCR) assay of nasal and pharyngeal swabs.

Critically ill patients were defined as those admitted to ICU who required mechanical ventilation or had a fraction of inspired oxygen (FiO_2_) of at least 60% or more [11]. Pregnant women, children (those younger than 18 years of age) and patients with two negative RT-PCR assay were excluded from the study. Informed consent was collected in only a small amount of patients due to the rapidly worsening of their clinical conditions. The ethics committee allowed this conduct since the early COVID-19pandemic phase has seriously hampered the ability to achieve a traditional informed consent before study enrolment.

### Data collection

We reviewed all the electronic medical records to collect demographic, clinical, laboratory and radiologic data from the hospital management software within the first 24 hours of ICU admission. The following laboratory variables were considered: complete blood cells count, C-reactive protein (CRP), creatinine, glucose, total bilirubin and procalcitonin.

Data from high-resolution chest computed tomography (CT) performed within 2 days of ICU admission were collected. CT lung pattern were defined as Patchy Ground-Glass Opacities (GGO), diffuse GGO, mixed consolidation + GGO or consolidation. The presence of bilateral lungs involvement, the visual assessment of lung involved percentage [12] and the presence of lung consolidation were also considered. Visual quantification was used to classify patients as the percentage of lung parenchyma affected by COVID-19 lesions. A radiologist with five years of experience performed the evaluation of each lung CT.

Patients’ clinical history including demographic data, medical comorbidities, Covid-19 symptoms duration before hospitalization were also collected. Lung protective ventilatory strategies were adopted and patients were treated according to current guidelines [13].

### Statistical analysis

A descriptive statistics was carried out. Continuous variables are reported as median and interquartile range while categorical data as relative number and percentage. Shapiro-Wilk test was used to test normality of distribution. We used the Mann-Whitney U test, χ2 test, or Fisher’s exact test to compare differences between survivors and non-survivors.

Potential predictors variables of 28-day mortality were firstly chosen based on their ease measurement during the ICU admission or for their previously showed role as mortality predictor [5, 14]. Due to the high clinical and radiological homogeneity of critically ill COVID-19 patients admitted to our ICU during the study period, we decided to use a Cox model to consider time-dependent covariates. A Kaplan-Meier survival estimates were used to evaluate the 28-day survival. The association of risk factors with 28 day-mortality was assessed in univariable and multivariable Cox proportional hazards regression models. The proportional hazard assumption was tested by plotting the Nelson-Aalen cumulative hazard function and Schoenfeld residuals test. A forward regression analysis was used to select variables accepted in the multivariable model. Factors for which p values were less than 0.1 in univariable analysis were used as candidate variables for multivariable approach. The Akaike information criterion was used to compare different regression models and to select the most parsimonious model.

Model performance was assessed via discrimination and calibration measures. To assess for discrimination, the C statistic was used. A calibration curve was implemented by comparing the predicted probabilities and the actually observed proportions, using the Stata module “pmcalplot” [15].

The TRIPOD (transparent reporting of a multivariable model for individual prognosis or diagnosis) guidance was used to conduct this study and to report the results of the prediction model [16].

For internal validation of the model, a non-parametric bootstrap (1000 replications) of the original model was run. The bootstrapped samples were created by drawing random samples with replacement from the development database. The prediction model was fitted on each of bootstrap samples. To adjust for optimism after model development, estimates of a uniform shrinkage factor (the average calibration slope from each of the bootstrap samples) were obtained and multiplied by the original β coefficients to obtain optimism adjusted hazard ratios for each variable [17].

Results are expressed as hazard ratio with 95% confidence intervals (95%CI) and p values. Statistical significance was set at a two tailed P value <0.05. STATA MP, version 16.0 (STATA Corp., Texas, USA) was used for the analysis.

## Results

**Fig 1:**
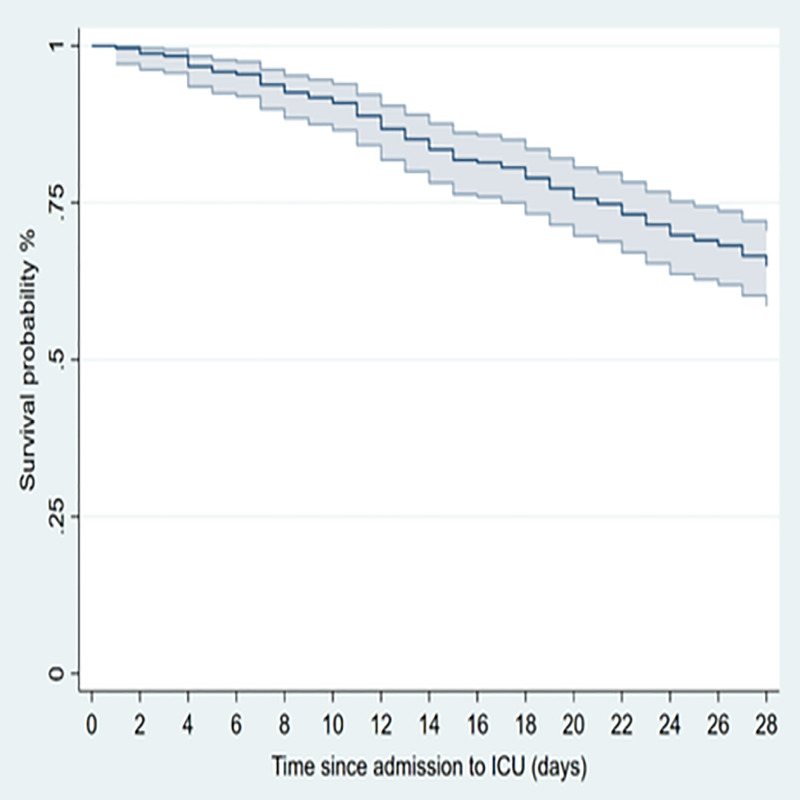
Survival of critically ill COVID-19 patients with pneumonia after the admission to the intensive care unit (ICU). Dashed lines represent 95% CIs.

242 patients with a confirmed SARS-CoV-2 infection were admitted to our ICU during the study period and represent the studied population. Two other patients were excluded due to negative RT-PCR findings for SARSCoV-2. The median age of the patients was 64 years (56–71 IQR) and 196 (81%) were male. Almost one comorbidity was present in 147 patients (61%) of which hypertension was the most common (46.7%), followed by diabetes (15.3%) and heart disease (14.5%), (Table 1). The most common findings at hospital admission were respiratory symptoms and fever (97.5% and 92.1% respectively), followed by gastrointestinal manifestations, mainly vomiting and diarrhea in 18.6%. Eighty-five patients (35.1%) died within 28 days after ICU admission and the median time from ICU admission to death was 11 days (IQR 6–18), (Fig 1).

**Table 1:** Demographic, clinical characteristic, comorbidities and outcomes of 242 patients with COVID-19 admitted to ICU. Data are reported as median, IQR and percentage of the total.

Demographic and clinical characteristics	Total (n = 242)	Non survivors (n = 85)	Survivors (n = 157)	p value
	n (%)	n (%)	n (%)	
**Time from symptom onset to hospital admission**, median (IQR), days	7 (6–10)	7 (6–10)	7 (7–10)	0.67
**Time from hospital admission to ICU admission**, median (IQR), days	4 (1–6)	4 (1–7)	4 (1–6)	0.69
**Age**, median (IQR), years	64 (56–71)	66 (60–73)	62 (55–69)	0.0002
**Gender**				
Female	46 (19%)	16 (18.9%)	30 (19.1%)	0.96
Male	196 (81%)	69 (81.2%)	127 (80.9%)	
**Comorbidities**				
Hypertension	113 (46.7%)	42 (49.4%)	71 (45.2%)	0.51
Coronary heart disease, atrial fibrillation	35 (14.5%)	14 (16.5%)	21 (13.4%)	0.48
Diabetes	37 (15.3%)	18 (21.2%)	19 (12.1%)	0.06
Obesity	31 (12.8%)	16 (18.8%)	15 (9.6%)	0.03
Chronic obstructive pulmonary disease	21 (8.7%)	10 (11.8%)	11 (7%)	0.21
Chronic kidney disease	6 (2.5%)	3 (3.5%)	3 (1.9%)	0.44
Malignancy or history of cancer disease	16 (6.6%)	6 (7%)	10 (6.4%)	0.79
**Initial symptoms**				
Fever	223 (92.1%)	76 (89.4%)	147 (93.6%)	0.23
Respiratory symptoms	236 (97.5%)	84 (98.8%)	152 (96.8%)	0.20
Cardiovascular symptoms	15 (6.2%)	7 (8.2%)	8 (5.1%)	0.41
Gastrointestinal symptoms	45 (18.6%)	18 (21.2%)	27 (17.2%)	0.46
**ICU length of stay**, median (IQR), days	3 (1–7)	7 (3–11)	2 (1–5)	<0.0001

The comparison of patients characteristics showed a higher prevalence of obesity (defined as BMI of at least 30 kg/m^2^) in the non-survivors compared to survivors (18.8% vs 9.6%, p = 0.03, respectively). Non-survivor patients were older than survivors, with a median age of 66 (60–73 IQR) years in non-survivors and 62 (55–69) years in survivors (p = 0.0002) (Table 1).

The median time from respiratory symptoms onset to hospital admission was not different in the two groups (7 days, 6–10 IQR in non-survivors vs 7 days, IQR 7–10 days in survivors, p = 0.67) as well as the length of hospital stay prior to ICU admission (4 days, 1–7 IQR in non-survivors vs 4 days, 1–6 IQR in survivors, p = 0.69).

At ICU admission, 26.9% of the patients were treated with C-PAP for almost 1 day (IQR 1–3) while 177 (73.1%) required immediate mechanical ventilation and 80 patients (35%) required the use of prone position ventilation. All the patients received dexamethasone 6mg once daily for 7–10 day while only five patients received compassionate-use remdesivir.

Laboratory findings at ICU admission are resumed in Table 2. Lymphocytopenia (< 1×10^9^/L) occurred in the totality of the patients and no difference was found between survivors and non-survivors (p = 0.84). Higher white blood cell (WBC) and neutrophils count was found in non-survivors compared to survivors (WBC 12.1 x 10^9^/L, IQR 8.2–18.5 in non-survivors and 10.3 x 10^9^/L, IQR 7.5–13.5 in survivors, p = 0.005; neutrophils 11 x 10^9^/L, IQR 7.2–17.3 in non-survivors and 9 x 10^9^/L, IQR 6.4–12.4 in survivors, p = 0.003). Non-survivor patients presented a reduced platelets count (p = 0.002) and increased procalcitonin levels (p = 0.001).

**Table 2:** Vital parameters, laboratory findings and treatments at ICU admission, data are reported as median, IQR.

Variables at ICU admission	Total (n = 242)	Non survivors (n = 85)	Survivors (n = 157)	p value
	n (%)	n (%)	n (%)	
**Vital parameters at ICU admission**, median (IQR)				
Heart rate, bpm	88 (75–102)	93 (75–110)	86 (74–96)	0.04
Respiratory rate, bpm	22 (18–27)	22 (18–24)	21 (19–30)	0.51
Mean arterial pressure, mmHg	72 (61–90)	81 (67–90)	81 (67–90)	0.33
PaO_2_/FiO_2_	112 (72–197)	88 (67–164)	134 (79–217)	0.005
**Laboratory indices at ICU admission**, median (IQR)				
White blood cells, x 10^9^/L	11.0 (7.8–15.8)	12.1 (8.2–18.5)	10.3 (7.5–13.5)	0.005
Neutrophils, x 10^9^/L	9.5 (6.6–14.2)	11 (7.2–17.3)	9 (6.4–12.4)	0.003
Lymphocytes, x 10^9^/L	0.6 (0.4–0.9)	0.6 (0.4–0.8)	0.6 (0.4–0.9)	0.84
Haemoglobin, g/dL	12.2 (11.2–13.3)	12.1 (11.2–13.4)	12.3 (11.4–13.2)	0.88
Platelets, x 10^9^/L	246 (181–320)	220 (165–268)	267 (195–333)	0.006
Total bilirubin, mg/dL	0.87 (0.64–1.47)	1.12 (0.64–1.87)	0.80 (0.64–1.19)	0.06
Urea, mg/dL	52 (39–71)	59 (42–85)	49 (38–63)	0.02
Creatinine, mg/dL	0.87 (0.66–1.17)	0.97 (0.74–1.23)	0.78 (0.64–1.06)	0.01
Procalcitonin, ng/mL	0.46 (0.16–1.39)	0.75 (0.31–1.77)	0.36 (0.11–1.01)	0.001
High-sensitivity C-reactive protein, mg/L	15.5 (8.35–23.63)	18.3 (10.24–26.78)	13.84 (6.81–22.61)	0.02
Glucose, mg/dL	169 (124–201)	130 (120–151)	130 (120–151)	0.14
**Arterial blood gas**				
pH	7.39 (7.32–7.45)	7.36 (7.28–7.41)	7.40 (7.33–7.46)	0.001
PaO_2_, mmHg	86 (66–146)	77 (62–112)	95 (70–166)	0.01
PaCO_2_, mmHg	44 (39–51)	49 (41–55)	43 (39–48)	0.0004
Lactate	12 (9–16)	13 (11–13)	11 (8–15)	0.004
**SOFA score**, median (IQR)	4 (3–5)	3 (3–4)	5 (4–6)	<0.001

High-resolution chest CT was performed in 229 (95%) patients. CT findings are summarized in Table 3. A consolidation + GGO pattern was the most common (53.7%) followed by diffuse GGO (24.9%). No difference in term of CT pattern was observed between survivors and non-survivors (p = 0.92). Bilateral lung involvement was present in 100% of the non-survivors and in the 97.3% of the survivors (p = 0.30). The extent of lesion on CT was very similar in the two groups and no differences were found (p = 0.69).

**Table 3:** Chest CT findings in survived and non-survived patients.

Chest CT findings	Total (n = 229)	Non survivors (n = 80)	Survivors (n = 149)	p value
	n (%)	n (%)	n (%)	
**CT pattern**				0.92
Patchy Ground-Glass Opacities	27 (11.8%)	10 (12.5%)	17 (11.4%)	
Diffuse Ground-Glass Opacities	57 (24.9%)	19 (23.8%)	38 (25.5%)	
Consolidation + Ground-Glass Opacities	123 (53.7%)	42 (52.5%)	81 (54.4%)	
Consolidation	22 (9.6%)	9 (11.2%)	13 (8.7%)	
**Bilateral lung involvement**	225 (98.3%)	80 (100%)	145 (97.3%)	0.30
**Extent of lesions on CT**				0.69
≤25%	30 (13%)	9 (11.2%)	21 (14.1%)	
25–50%	125 (54.6%)	47 (58.8%)	78 (52.3%)	
>50%	74 (32.4%)	24 (30%)	50 (33.6%)	

Check of the proportionality assumption before regression revealed no violation (χ^2^ = 10.02, p = 0.53). At univariable analysis diabetes (HR 1.80, 95% CI 1.07–3.04, p = 0.03), age (HR 1.05, 95% CI 1.02–1.08, p = <0.001), obesity (HR 1.99, 95% CI 1.15–3.45, p = 0.01), WBC count (HR 1.06, 95% CI 1.03–1.10, p<0.001), neutrophils count (HR 1.07, 95% CI 1.03–1.11, p<0.001), high-sensitivity C-reactive protein value (HR 1.03, 95% CI 1.00–1.05, p = 0.02), procalcitonin (HR 1.04, 95% CI 1.01–1.06, p = 0.002), SOFA score (HR 1.78, 95% CI 1.57–2.02, p<0.001), lactate (HR 1.02, 95% CI 1.00–1.04, p = 0.02), PaO_2_/FiO_2_ (HR 0.99, 95% CI 0.98–0.99, p<0.001) were associated with 28-day mortality.

The final internal validated multivariable model included age (optimism adjusted HR 1.04, 95% CI 0.99–1.09, p = 0.003), obesity (optimism adjusted HR 2.35, 95% CI 1.29–4.23, p = 0.04), procaltitonin (optimism adjusted HR 1.03, 95% CI 0.87–1.22, p = 0.04), SOFA score (optimism adjusted HR 1.40, 95% CI 0.99–1.99, p = 0.003) and PaO_2_/FiO_2_ (optimism adjusted HR 0.87, 95% CI 0.84–0.98, p = 0.003), (Table 4).

**Table 4:** Multivariable Cox proportional hazards regression analysis of factors associated with mortality.

Variable	HR	95% CI	p value	Optimism adjusted HR	Optimism adjusted 95% CI
**Age**	1.05	1.00–1.08	0.003	1.04	0.99–1.09
**Diabetes**	1.13	0.46–2.77	0.79	1.43	0.77–2.36
**Obesity**	2.33	1.03–5.26	0.04	2.35	1.29–4.23
**White blood cells, x 109/L**	1.25	0.89–1.75	0.20	1.26	0.77–2.06
**High-sensitivity C-reactive protein, mg/L**	1.03	0.99–1.06	0.08	1.02	1.02–1.07
**Procalcitonin**	1.03	1.01–1.06	0.04	1.03	0.87–1.22
**SOFA**	1.37	1.11–1.69	0.003	1.40	0.99–1.99
**Neutrophils, x 10^9^/L**	0.79	0.54–1.14	0.21	0.78	0.46–1.32
**Urea, mg/dL**	1.01	0.99–1.02	0.06	1.01	0.99–1.02
**Lactate**	0.98	0.96–1.01	0.28	0.99	0.95–1.04
**PaO_2_/FiO_2_**	0.88	0.86–0.99	0.003	0.87	0.84–0.98

**Fig 2:**
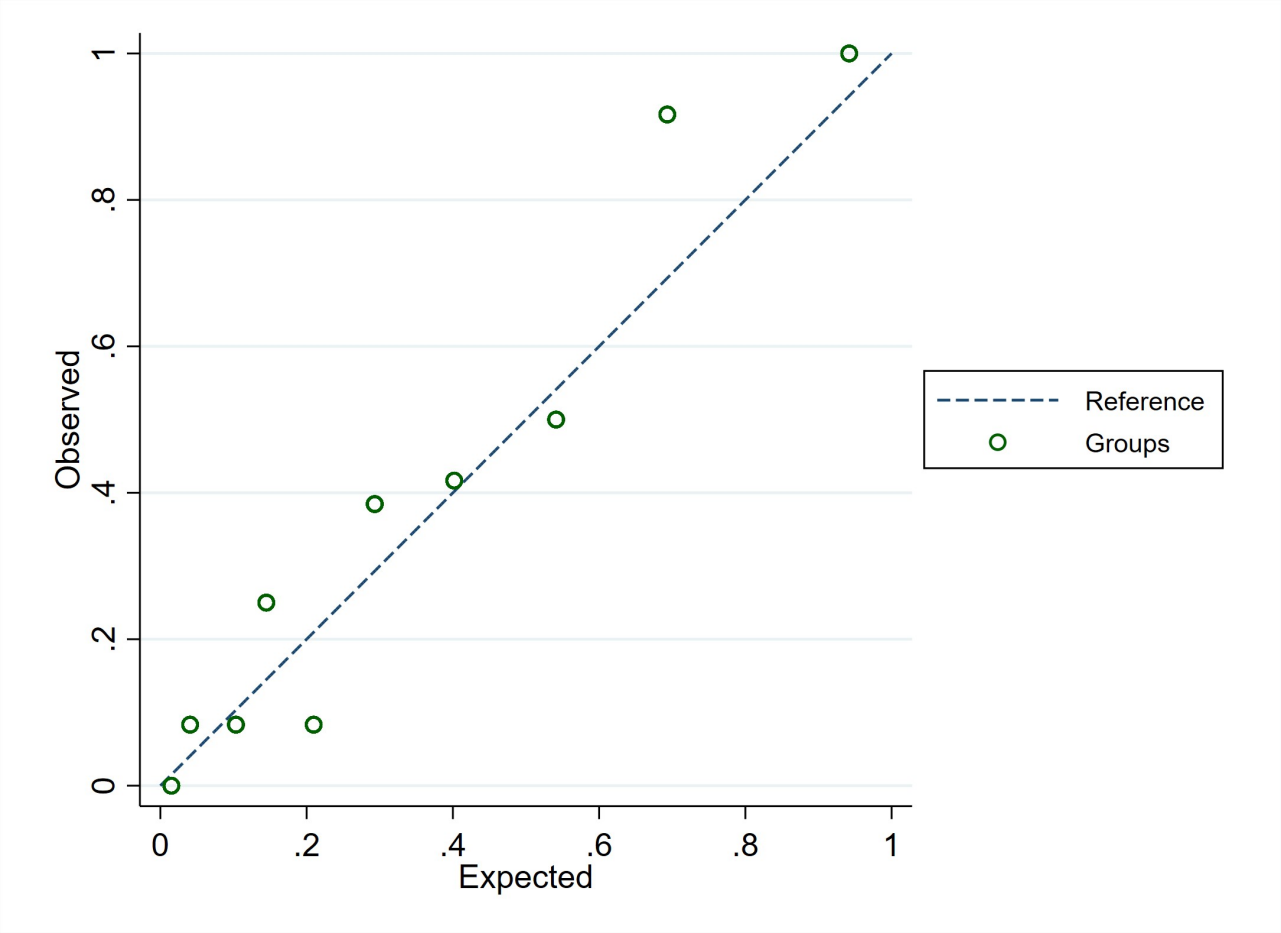
Calibration plot of the multivariable prediction model for 28-day mortality of critically ill COVID-19 patients admitted to ICU.

The C-statistic for the predicted 28-day mortality risk showed very good discriminatory capacity equal to 0.821 (95% CI 0.766–0.876) and 0.822 (95% CI 0.770–0.873) in the original and bootstrap models, respectively. The estimated bias was 1.1*10^−3^ (95% CI 1.9*10^−2–^3.4*10^−3^). The calibration plot revealed good agreements between the observed and expected probability of death (Fig 2).

## Discussion

The COVID-19 pandemic is a worldwide novel challenge for critical care systems since it has strongly proved ICU capacities.

In the present study, multivariable Cox proportional hazards regression identified several prognostic markers for 28-day mortality. After adjustment for other factors, age, obesity, procalcitonin, SOFA and PaO_2_/FiO_2_ were independently associated with 28-day mortality in critically ill COVID-19 patients.

The majority of our patients (73%) were admitted to the ICU because of acute hypoxemic respiratory failure that required mechanical ventilation. The need for mechanical ventilation among COVID-19 patients admitted to ICUs ranges from 29.1% in one Chinese study [18] to 89.9% in a U.S. study [19] and 88% in an Italian study [8]. In this context, several studies have investigated the factors associated with death or ICU admission but limited information exist in Italian population for the prognostic factors associated with mortality in critically ill COVID-19 patients.

The ICU worldwide mortality for COVID-19 respiratory failure is 25.7% [20]. In Italy, a rate of 25.6% [8] was initially reported but a few months later the same authors reported a mortality rate of 48.7% in a subsequent study [9]. In the current study, the 28-day mortality rate of COVID-19 critically ill patients was 35.1%. This data is close to the average of the two previously mentioned mortality rates and it is similar to what was reported for ARDS [21]. However, as Quah outlined it in a letter to editor, almost a half of patients were still in ICU when previous studies of ICU mortality were published [20]. On the contrary, at the time of writing this paper, no patients were still in ICU as they were deceased or discharged. This element increase the meaning of our data since all the patients concluded the 28-day follow-up.

In our study, the patients were mainly middle-aged men with hypertension and the most common initial symptoms were fever and respiratory symptoms such as cough and dyspnea. These findings are in concordance with previously published studies [7, 8, 22, 23]. In fact, the age was independently associated with an increased risk of 28-day mortality in the multivariable analysis. This is not unexpected since this variable was extensively associated with adverse outcome [9, 24, 25]. Obesity was another important predictor of 28-day mortality (p = 0.04). This finding seems related to the chronic inflammatory state in obese patients [26] that can increase the excessive cytokine response to viral infection leading to adverse outcome [27, 28]. As we previously reported, obesity and morbid obesity were risk factors for death in critically ill COVID-19 patients [29].

An increase in procalcitonin levels was reported as an indicator of disease severity in COVID-19 patients [30] and as a risk factor for mortality [31]. Our findings seems in line with these evidences since an increase in procalcitonin levels is associated with increased 28-day mortality rates at multivariable analysis (p = 0.04).

Interestingly, in our patients the presence of diabetes was not associated with an increased mortality risk. This is in contrast with many case series reporting that people with diabetes are at higher risk of COVID-19-related mortality [32] and ICU admission with poor outcome than people without diabetes [33]. However, some factors rarely considered such as type of diabetes, length of the disease and related complications, type of treatment and glycaemic controls during the infection could have affected the outcome.

The lymphocytopenia observed in our patients confirms that they were critically ill COVID-19 patients since a low lymphocyte count is related to the severity of disease [34].

Higher Sequential Organ Failure Assessment (SOFA) score on admission was independently associated with an increased 28-day mortality risk (HR 1.37, 95% CI 1.01–1.05, p = 0.04). In fact, as it was previously published, this score is a highly sensitive marker of in-hospital mortality in COVID-19 patients and can be considered as a risk-stratification tool for critical COVID-19 patients [35]. Moreover, a low PaO_2_/FiO_2_ ratio at ICU admission was an independent risk factor associated with 28-day mortality. This is not unexpected since acute respiratory failure is the leading feature in critical COVID-19 patients.

Even if male are at higher risk for mortality in the overall population, any difference in mortality rate could not be demonstrated between male and female patients once admitted to the ICU as previously reported by Nachtigall in a large group of critically ill COVID-19 patients hospitalized in Germany [36].

As it was previously published, a lung involvement >50% is associated with ICU admission in hospitalized patients [37]. On the other hand, patients with well-aerated lung parenchyma less than 73% are at increased risk for ICU admission or death [38]. Interestingly, in our patients, the radiological findings at chest CT were approximately the same in survivors and non-survivors and no predictive ability was found. Since the median time from symptom onset to the ICU admission as well as the length of hospital stay prior to ICU admission were similar in survivors and in non-survivors (p = 0.67) it is possible to argue that the observed lung abnormalities are related to the disease time course. Consequently, in our study it is not possible to verify the rule of radiological data to predict mortality.

A high percentage of patients required mechanical ventilation since ICU admission and the principal reason for ICU admission was related to the severity of respiratory failure. We can suppose that disease severity was similar in survivors and in non-survivors. Therefore, due to the characteristics of our sample a validation of the clinical prediction model is needed.

Many prognostication model for patients with COVID-19 were recently developed but a possible risk of bias was outlined due to the limited use of validation techniques [39]. For these reasons, we decided to perform an internal validation to prevent model over-fitting and to obtain a reduction of potential false positive prediction estimates. The bootstrap technique was used since it provides stable estimates with low bias of predictors [40] and it is considered as a central technique to correct overfitting and to quantify optimism in model performance [41].

To our knowledge, this is the first model of 28-day mortality in critically ill COVID-19 patients that was internal validated and it is the third study about critically ill COVID-19 patients requiring ICU admission in Italy. Moreover, our patients were admitted to ICU in a geographic area that is one of the first place where the COVID-19 outbreak spread across Italy.

Our results were obtained from the first phase of the COVID-19 pandemic in Italy and helped us to improve our healthcare organization for the second pandemic phase. However, this model should be confirmed in the subsequent phases of this pandemic.

Nevertheless, this study has several limitations. First, it is a retrospective study during a pandemic that overwhelmed the medical resources. Therefore, it is possible that some data present inaccuracies that might introduce some bias in the study results. Second, we did not collect data about complications during ICU length of stay such as secondary infection, organ failure or thrombosis. Thrombosis and thromboembolism have been reported as a relevant topic in COVID-19 critically ill patients with a prevalence up to 30% but these data were not clearly known at the time we admitted our patients to ICU. The same problem can be applied to D-dimer and IL-6 that we did not collect in every patient. Third, we did not collect the cause of death in our patients with COVID-19 but it is possible to suppose that hypoxemia was the leading cause of death. Forth, no data were collected about type of mechanical ventilation used along with pulmonary compliance and resistance even if two possible respiratory patterns were described [42] and a possible role in mortality rate could be supposed. Another major limit is the absence of an external validation for our model that we hope to perform in a subsequent cohort of COVID-19 critically ill patients.

## Conclusions

In this study we developed an internal validated prediction model for 28-day mortality of critically ill COVID-19 patients admitted to ICU with the use of simple and easy to collect clinical variables. Age, obesity, procalcitonin, SOFA score and PaO_2_/FiO_2_ ratio emerged as independent predictors for mortality and should be carefully evaluated in these patients. We hope that these data might help a better organization of ICUs for the treatment of COVID-19 patients. Future studies with increased patients number and longer follow-up are needed to confirm our findings.

## References

[1] Team NCPERE. Vital surveillances: the epidemiological characteristics of an outbreak of 2019 novel coronavirus diseases (COVID-19)–China. China CDC Weekly 2020; 2(8):113–122. PMC8392929 34594836

[2] Covid-19 coronavirus pandemic. Updated February 4, 2021. Accessed March 15, 2021. https://www.worldometers.info/coronavirus

[3] Guan WJ, Ni ZY, Hu Y, et al. Clinical Characteristics of Coronavirus Disease 2019 in China. N Engl J Med 2020; 382: 1708–1720. doi: 10.1056/NEJMoa2002032 32109013 PMC7092819

[4] Poggiali E, Vercelli A, Mazzoni S, Bastoni D, Iannicelli T, Demichele E et al. COVID-19 pandemic, Piacenza calling. The survival strategy of an Italian Emergency Department. Acta Biomed. 2020 Jun 4;91(3). doi: 10.23750/abm.v91i3.9908 32921738 PMC7717017

[5] Wang D, Hu B, Hu C, et al. Clinical characteristics of 138 hospitalized patients with 2019 novel coronavirus-infected pneumonia in Wuhan, China. JAMA. 2020. doi: 10.1001/jama.2020.1585 32031570 PMC7042881

[6] Wang Y, Lu X, Li Y, et al. Clinical course and outcomes of 344 intensive care patients with COVID-19. Am J Respir Crit Care Med. 2020;201(11):1430–1434. doi: 10.1164/rccm.202003-0736LE 32267160 PMC7258632

[7] Richardson S, Hirsch JS, Narasimhan M, et al; and the Northwell COVID-19 Research Consortium. Presenting characteristics, comorbidities, and outcomes among 5700 patients hospitalized with COVID-19 in the New York City area. JAMA. 2020;323(20):2052–2059. doi: 10.1001/jama.2020.6775 32320003 PMC7177629

[8] Grasselli G, Zangrillo A, Zanella A, et al; COVID-19 Lombardy ICU Network. Baseline characteristics and outcomes of 1591 patients infected with SARS-CoV-2 admitted to ICUs of the Lombardy Region, Italy. JAMA. 2020;323(16):1574–1581. doi: 10.1001/jama.2020.5394 32250385 PMC7136855

[9] Grasselli G, Greco M, Zanella A, et al; and the COVID-19 Lombardy ICU Network. Risk Factors Associated With Mortality Among Patients With COVID-19 in Intensive Care Units in Lombardy, Italy. JAMA Intern Med. 2020 Jul 15;e203539. doi: 10.1001/jamainternmed.2020.3539 32667669 PMC7364371

[10] Clinical management of severe acute respiratory infection when novel coronavirus (‎‎‎‎‎‎2019-nCoV)‎‎‎‎‎‎ infection is suspected: interim guidance, 13 March 2020.

[11] Kumar A, Zarychanski R, Pinto R, et al. Critically ill patients with 2009 influenza A(H1N1) infection in Canada. JAMA 2009;302: 1872–79 doi: 10.1001/jama.2009.1496 19822627

[12] Yu N, Shen C, Yu Y, Dang M et al. Lung involvement in patients with coronavirus disease-19 (COVID-19): a retrospective study based on quantitative CT findings. Chin J Acad Radiol. 2020 May 11: 1–6. doi: 10.1007/s42058-020-00034-2 32395696 PMC7211979

[13] COVID-19 Treatment Guidelines Panel. Coronavirus Disease 2019 (COVID-19) Treatment Guidelines. National Institutes of Health. Available at https://www.covid19treatmentguidelines.nih.gov/. 34003615

[14] Huang C, Wang Y, Li X, et al. Clinical features of patients infected with 2019 novel coronavirus in Wuhan, China. Lancet 2020; 395: 497–506. doi: 10.1016/S0140-6736(20)30183-5 31986264 PMC7159299

[15] Ensor J, Snell KI, Martin EC. PMCALPLOT: Stata module to produce calibration plot of prediction model performance. Stat Softw Components 202

[16] Moons KG, Altman DG, Reitsma JB, et al. Transparent Reporting of a multivariable prediction model for Individual Prognosis or Diagnosis (TRIPOD): explanation and elaboration. Ann Intern Med 2015;162:W1–73. doi: 10.7326/M14-0698 25560730

[17] Van Houwelingen JC, Le Cessie S. Predictive value of statistical models. Stat Med 1990;9:1303–25. doi: 10.1002/sim.4780091109 2277880

[18] Wang Y, Lu X, Li Y, Chen H, Chen T, Su N, et al. Clinical Course and Outcomes of 344 Intensive Care Patients with COVID-19. Am J Respir Crit Care Med. 2020 Jun 1;201(11):1430–1434. doi: 10.1164/rccm.202003-0736LE 32267160 PMC7258632

[19] Richardson S, Hirsch JS, Narasimhan M, Crawford JM, McGinn T, Davidson KW, et al. Presenting characteristics, comorbidities, and outcomes among 5700 patients hospitalized with COVID-19 in the New York city area. JAMA. 2020;323:2052–2059. doi: 10.1001/jama.2020.6775 32320003 PMC7177629

[20] Quah P, Li A, Phua J. Mortality rates of patients with COVID-19 in the intensive care unit: a systematic review of the emerging literature. Crit Care. 2020 Jun 4;24(1):285. doi: 10.1186/s13054-020-03006-1 32498689 PMC7271132

[21] Máca J, Jor O, Holub M, Sklienka P, Burša P et al. Past and Present ARDS Mortality Rates: A Systematic Review. Respir Care. 2017 Jan;62(1):113–122. doi: 10.4187/respcare.04716 Epub 2016 Nov 1. 27803355

[22] Zhou F, Yu T, Du RH, et al. Clinical course and risk factors for mortality of adult inpatients with COVID-19 in Wuhan, China: a retrospective cohort study. Lancet 2020;395(10229):1054–62. doi: 10.1016/S0140-6736(20)30566-3 32171076 PMC7270627

[23] Huang CL, Wang YM, Li XW, et al. Clinical features of patients infected with 2019 novel coronavirus in Wuhan, China. Lancet 2020;395(10223):497–506. doi: 10.1016/S0140-6736(20)30183-5 31986264 PMC7159299

[24] Wang J, Zhang J, Tu Y, Zhou X, Huang H, Shao L, et al. Cancer patients in SARS-CoV-2 infection: a single-center experience from Wuhan. J Cancer. 2020 Aug 27;11(21):6243–6247. doi: 10.7150/jca.47065 ; PMCID: PMC7532501. 33033507 PMC7532501

[25] Levy TJ, Richardson S, Coppa K, Barnaby DP, McGinn T, Becker LB, et al. Development and Validation of a Survival Calculator for Hospitalized Patients with COVID-19. medRxiv [Preprint]. 2020 Apr 27:2020.04.22.20075416. doi: 10.1101/2020.04.22.20075416 32511640 PMC7276996

[26] Holly JMP, Biernacka K, Maskell N, Perks CM. Obesity, Diabetes and COVID-19: An Infectious Disease Spreading From the East Collides With the Consequences of an Unhealthy Western Lifestyle. Front Endocrinol (Lausanne). 2020 Sep 17;11:582870. doi: 10.3389/fendo.2020.582870 ; PMCID: PMC7527410. 33042029 PMC7527410

[27] Rumende CM, Susanto EC, Sitorus TP. The Management of Cytokine Storm in COVID-19. Acta Med Indones. 2020 Jul;52(3):306–313. . 33020343

[28] Nakeshbandi M, Maini R, Daniel P, Rosengarten S, Parmar P, Wilson C, et al. The impact of obesity on COVID-19 complications: a retrospective cohort study. Int J Obes (Lond). 2020 Sep;44(9):1832–1837. doi: 10.1038/s41366-020-0648-x Epub 2020 Jul 25. ; PMCID: PMC7382318. 32712623 PMC7382318

[29] Halasz G, Leoni ML, Villani GQ, Nolli M, Villani M. Obesity, overweight and survival in critically ill patients with SARS-CoV-2 pneumonia: is there an obesity paradox? Preliminary results from Italy. Eur J Prev Cardiol. 2020 Jul 7:2047487320939675. 10.1177/2047487320939675 PMC7928993 32635756

[30] Hu R, Han C, Pei S, Yin M, Chen X. Procalcitonin levels in COVID-19 patients. Int J Antimicrob Agents. 2020 Aug;56(2):106051. doi: 10.1016/j.ijantimicag.2020.106051 Epub 2020 Jun 10. ; PMCID: PMC7286278. 32534186 PMC7286278

[31] Liu ZM, Li JP, Wang SP, Chen DY, Zeng W, Chen SC, et al. Association of procalcitonin levels with the progression and prognosis of hospitalized patients with COVID-19. Int J Med Sci. 2020 Sep 9;17(16):2468–2476. doi: 10.7150/ijms.48396 ; PMCID: PMC7532477. 33029089 PMC7532477

[32] Barron E, Bakhai C, Kar P, Weaver A, Bradley D, Ismail H, et al. Associations of type 1 and type 2 diabetes with COVID-19-related mortality in England: a whole-population study. Lancet Diabetes Endocrinol. 2020 Oct;8(10):813–822. doi: 10.1016/S2213-8587(20)30272-2 Epub 2020 Aug 13. PMCID: PMC7426088. 32798472 PMC7426088

[33] Roncon L, Zuin M, Rigatelli G, Zuliani G. Diabetic patients with COVID-19 infection are at higher risk of ICU admission and poor short-term outcome. J Clin Virol. 2020 Jun;127:104354. doi: 10.1016/j.jcv.2020.104354 Epub 2020 Apr 9. ; PMCID: PMC7195018. 32305882 PMC7195018

[34] Yang L, Liu J, Zhang R, Li M, Li Z, Zhou X, et al. Epidemiological and clinical features of 200 hospitalized patients with corona virus disease 2019 outside Wuhan, China: A descriptive study. J Clin Virol. 2020 Aug;129:104475. doi: 10.1016/j.jcv.2020.104475 Epub 2020 May 26. ; PMCID: PMC7250074. 32485619 PMC7250074

[35] Liu S, Yao N, Qiu Y, He C. Predictive performance of SOFA and qSOFA for in-hospital mortality in severe novel coronavirus disease. Am J Emerg Med. 2020 Jul 12. doi: 10.1016/j.ajem.2020.07.019 Epub ahead of print. PMCID: PMC7354270. 33142178 PMC7354270

[36] Nachtigall I, Lenga P, Jóźwiak K, Thürmann P, Meier-Hellmann A, Kuhlen R, et al. Clinical course and factors associated with outcomes among 1904 patients hospitalized with COVID-19 in Germany: an observational study. Clin Microbiol Infect. 2020 Aug 18:S1198-743X(20)30493-6. doi: 10.1016/j.cmi.2020.08.011 32822883 PMC7434317

[37] Ruch Y, Kaeuffer C, Ohana M, Labani A, Fabacher T, Bilbault P, et al. CT lung lesions as predictors of early death or ICU admission in COVID-19 patients. Clin Microbiol Infect. 2020 Oct;26(10):1417.e5–1417.e8. doi: 10.1016/j.cmi.2020.07.030 Epub 2020 Jul 24. 32717417 PMC7378475

[38] Colombi D, Bodini FC, Petrini M, Maffi G, Morelli N, Milanese G, et al. Well-aerated Lung on Admitting Chest CT to Predict Adverse Outcome in COVID-19 Pneumonia. Radiology. 2020 Aug;296(2):E86–E96. doi: 10.1148/radiol.2020201433 Epub 2020 Apr 17. ; PMCID: PMC7233411. 32301647 PMC7233411

[39] Wynants L, Van Calster B, Collins GS, Riley RD, Heinze G, Schuit E, et al. Prediction models for diagnosis and prognosis of covid-19 infection: systematic review and critical appraisal. BMJ. 2020 Apr 7;369:m1328. doi: 10.1136/bmj.m1328 Erratum in: BMJ. 2020 Jun 3;369:m2204. ; PMCID: PMC7222643. 32265220 PMC7222643

[40] Steyerberg EW, Harrell FE Jr, Borsboom GJ, Eijkemans MJ, Vergouwe Y, Habbema JD. Internal validation of predictive models: efficiency of some procedures for logistic regression analysis. J Clin Epidemiol. 2001 Aug;54(8):774–81. doi: 10.1016/s0895-4356(01)00341-9 . 11470385

[41] Tantithamthavorn C., McIntosh S., Hassan A.E., Matsumoto K.: An empirical comparison of model validation techniques for defect prediction models. IEEE Transactions on Software Engineering 43, 1–18 (2017).

[42] Gattinoni L, Chiumello D, Caironi P, Busana M, Romitti F, Brazzi L, et al. COVID-19 pneumonia: different respiratory treatments for different phenotypes? Intensive Care Med. 2020 Jun;46(6):1099–1102. doi: 10.1007/s00134-020-06033-2 Epub 2020 Apr 14. ; PMCID: PMC7154064. 32291463 PMC7154064

